# Comprehensive Individualized Person‐Centered Management Program Reduces Psychotropic Medication Costs and the Behavioral and Psychological Symptoms of Dementia in Advanced Alzheimer's Persons in a 28‐Week Randomized Controlled Trial

**DOI:** 10.1002/alz70858_107125

**Published:** 2025-12-26

**Authors:** Sunnie Kenowsky, Yongzhao Shao, Sloane Heller, James B. Golomb, Qiao Zhang, Gaurav Vedvyas, Gianna Dafflisio, Karyn Marsh, Martin Sadowski, Thomas Wisniewski, Arjun V. Masurkar, Barry Reisberg

**Affiliations:** ^1^ Center for Cognitive Neurology, New York University Langone Health, New York, NY, USA; ^2^ New York University Grossman School of Medicine, New York, NY, USA; ^3^ Alzheimer's Disease Research Center, New York University Langone Health, New York, NY, USA; ^4^ Division of Critical Care and Hospital Neurology, Columbia University Irving Medical Center, New York, NY, USA; ^5^ New York University Langone Health, New York, NY, USA; ^6^ Penn State College of Medicine, Penn State Health Milton S. Hershey Medical Center, Hershey, PA, USA; ^7^ McGill University Centre for Studies in Aging, Montreal, QC, Canada

## Abstract

**Background:**

Behavioral and psychological symptoms of dementia (BPSD) occur frequently and cause suffering, burden, and institutionalization in Alzheimer's disease affected persons. Medications prescribed to treat BPSD are expensive and often are not highly effective. Brexpiprazole recently approved to treat Alzheimer's agitation carries a retail drug price of $79.50/pill. Acetylcholinesterase inhibitors (ACHI) have been found to ameliorate BPSD in Alzheimer's persons in some studies. However, they are not commonly prescribed for BPSD by community physicians. We published results of a 28‐week, clinician‐blind, randomized, controlled trial of our comprehensive, individualized person‐centered management (CI‐PCM) program in moderate‐to‐severe, community‐residing Alzheimer's persons receiving memantine (doi:10.1159/000455397). We examined BPSD using the Behavioral Pathology in Alzheimer's Disease Frequency Weighted Severity Scale, which showed significantly decreased BPSD in the CI‐PCM group at 28‐weeks (*p* <0.05). Additionally, the total amount of psychotropic medication prescribed to treat BPSD was lower in CI‐PCM subjects at 28‐weeks in comparison to baseline (*p* <0.0001, https://doi.org/10.1002/alz.056048). Herein, we examine if CI‐PCM subjects in our 2017 trial benefited from psychotropic medication cost savings and whether differential ACHI usage accounted for improvements in BPSD in the CI‐PCM group.

**Methods:**

Medication usage was captured at each study visit. Blinded investigators reviewed subject charts (*N* = 20), for total psychotropic drug usage and total psychotropic medication prescribed to treat BPSD. Retail drug pricing of each prescription was obtained from the NYU outpatient pharmacy to standardize costs. Total cost of psychotropic medication prescribed to treat BPSD was calculated for each individual and study group at baseline and weeks 4,12, and 28. Differences between groups were compared using the Wilcoxon Sum Rank test.

**Results:**

There were no statistically significant differences between groups in ACHI used or not (*p* = 0.401), number of days on and days off ACHI (*p* = 0.58) or in costs of psychotropic medication taken. CI‐PCM subjects paid $8,804.69 and Comparator subjects paid $24,901 from baseline to week 28 to treat BPSD.

**Conclusions:**

CI‐PCM subjects saved an average of $1,609.63 and experienced improvements in BPSD whereas comparators spent 2.8 times more and had worsening BPSD. ACHI treatment did not differ significantly between the two groups or account for the improvement in CI‐PCM group BPSD.

